# Clinical Validation of GenBody COVID-19 Ag, Nasal and Nasopharyngeal Rapid Antigen Tests for Detection of SARS-CoV-2 in European Adult Population

**DOI:** 10.3390/biomedicines11020493

**Published:** 2023-02-08

**Authors:** Karolina Wegrzynska, Jaroslaw Walory, Radoslaw Charkiewicz, Marzena Anna Lewandowska, Izabela Wasko, Aleksandra Kozinska, Piotr Majewski, Anna Baraniak

**Affiliations:** 1 Department of Biomedical Research, National Medicines Institute, 00-725 Warsaw, Poland; 2 Department of Clinical Molecular Biology, Medical University of Bialystok, 15-269 Bialystok, Poland; 3 Center of Experimental Medicine, Medical University of Bialystok, 15-369 Bialystok, Poland; 4 The F. Lukaszczyk Oncology Center, Molecular Oncology and Genetics Department, Innovative Medical Forum, 85-796 Bydgoszcz, Poland; 5 Department of Thoracic Surgery and Tumors, Ludwik Rydygier Collegium Medicum in Bydgoszcz, Nicolaus Copernicus University, 85-067 Torun, Poland; 6 Department of Microbiological Diagnostics and Infectious Immunology, Medical University of Bialystok, 15-369 Bialystok, Poland

**Keywords:** clinical trial, clinical validation, GenBody COVID-19 Ag, rapid antigen test (RAT), RT-qPCR

## Abstract

Accurate and rapid identification of COVID-19 is critical for effective patient treatment and disease outcomes, as well as the prevention of SARS-CoV-2 transmission. Rapid antigen tests (RATs) for identifying SARS-CoV-2 are simpler, faster and less expensive than molecular assays. Any new product to be considered a medical device is subject to evaluation and data analysis to verify the in vitro diagnostic ability to achieve its intended purpose. Clinical validation of such a test is a prerequisite before clinical application. This study was a clinical validation on adult Europeans of GenBody COVID-19 Ag, nasal and nasopharyngeal RATs. A set of 103 positive and 301 negative from nose and nasopharynx samples confirmed by RT-qPCR were examined. The tests were safe to use and showed 100% specificity in both specimens, and high sensitivity of 94.17% (95%CI 87.75% to 97.83%) and 97.09% (95%CI 91.72% to 99.4%), respectively. The parameters were significantly better for samples with higher virus loads (the highest for CT ≤ 25). The GenBody COVID-19 Ag RATs are inexpensive (compared to RT-qPCR), reliable and rapid with high sensitivity and specificity, making them suitable for diagnosis and timely isolation and treatment of COVID-19 patients, contributing to the better control of virus spread.

## 1. Introduction

The worldwide spread of the novel severe acute respiratory syndrome coronavirus 2 (SARS-CoV-2) and the resulting coronavirus disease 2019 (COVID-19) constitute a major public health challenge. Just a few months after the disease was first identified, it was granted pandemic status by the World Health Organization [1]. The clinical spectrum of SARS-CoV-2 infection ranges from asymptomatic or mild symptoms to severe pneumonia and acute respiratory distress syndrome, which can lead to death [2,3,4,5]. The course of the illness is influenced by many factors, including individual characteristics (age, gender, comorbidities and host genetic factors), contextual aspects (social determinants and organizational issues such as the burden on hospitals), the variant of the virus causing infection (new variants possess mutations affecting their transmissibility and immune escape), and finally the timing of diagnosis and the applied treatment [6,7,8,9]. Therefore, the identification of SARS-CoV-2 can be crucial to patient prognosis and disease outcome. In addition, the detection of every infected person, including asymptomatic ones, and the application of isolation leads to a reduction in the spread of the virus and, consequently, to the control of the pandemic.

**Figure 1:**
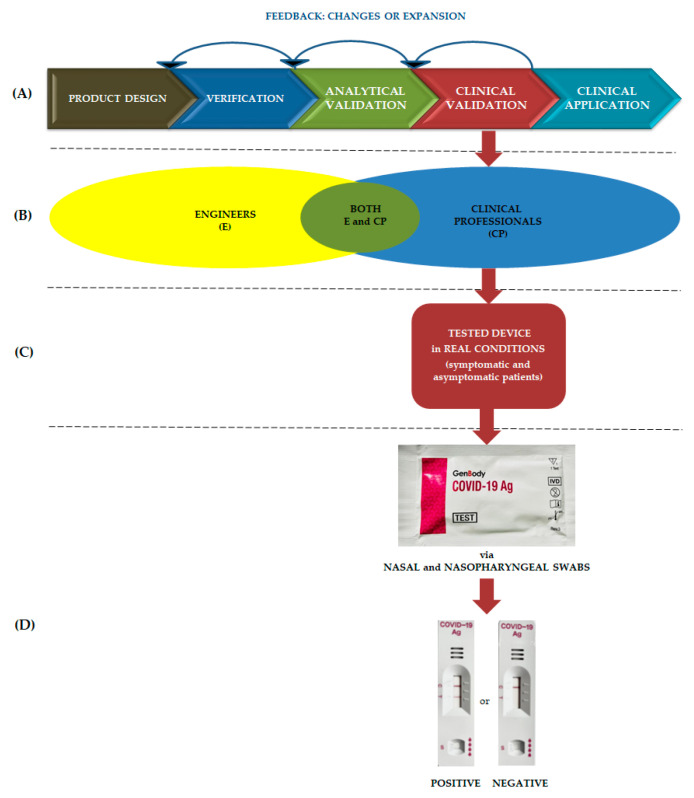
General view of the clinical trial: (**A**) stages including checkpoints, (**B**) role of various disciplinary experts, (**C**) device evaluation in clinical conditions, (**D**) tested medical device including visualization of positive and negative results.

As SARS-CoV-2 is a novel virus in the human population, there was a need to adopt known tools or develop new tests to detect the virus for rapid and reliable diagnostics and to monitor its dissemination. New products to be considered medical devices are subject to evaluation and data analysis in accordance with the ISO 13485 standard to establish/verify their in vitro diagnostic capability to achieve the intended clinical application [10]. A general overview of a clinical trial of a medical device based on Goldsack et al. [11] is shown in Figure 1. It consists of verification, followed by analytical and clinical validation, which leads to the determination of the sensitivity and specificity of the test. At each step of the evaluation, there is feedback to an earlier level about the obtained results, so that any changes can be implemented. Verification provides the clinical user information on how to apply a particular test, while analytical validation focuses on the performance of the test and its ability to achieve the intended purpose, such as the detection of coronaviruses. Finally, clinical validation is a process that evaluates whether a test device acceptably identifies a patient’s clinical condition and is useful in practice.

According to the European Union (EU) regulations, any new assay to be included in the common list of COVID-19 tests [12] must undergo evaluation and meet the criteria of the SARS-CoV-2 in vitro diagnostic (IVD) medical device performance evaluation guidelines [13,14]. Clinical validation should be carried out in an independent laboratory of the EU member state and conducted as a prospective investigation with unselected symptomatic and asymptomatic participants. The target study population should include at least 100 positive and 300 negative samples confirmed by the reference method. There are currently 78 RATs on the EU COVID-19 list of common diagnostic tests, and their parameters (sensitivity/specificity) are publicly available [14].

Two main approaches are used in testing for COVID-19, one based on the identification of structural components of the virus and the other detecting antibodies produced by the host immune system in response to SARS-CoV-2 infection [15]. However, using the former strategy allows the detection of an active infection in both symptomatic and asymptomatic individuals. It includes methods based on the identification of RNA (e.g., reverse transcription-quantitative polymerase chain reaction, RT-qPCR) or viral proteins (e.g., antigen tests) [15,16]. The specific and highly sensitive RT-qPCR assay was available almost from the outset of the pandemic and still remains the gold standard for the identification of SARS-CoV-2 by medical laboratories [17]. However, it was the rapid antigen tests (RATs), which detect specific viral antigens using lateral flow techniques, that dominated the market of diagnostic assays identifying the virus due to the short examination time, ease of performance, and lack of specialized equipment. Although the guidelines for their application are strictly defined and the sensitivity and specificity lower than the reference method, they are eagerly used for primary case detection, contact tracing, during outbreak investigations and to monitor trends of disease incidence in communities [18,19,20,21].

The aim of the study was to verify the clinical efficacy of GenBody COVID-19 Ag RATs compared to the reference method (RT-qPCR) by clinical validation of mostly prospectively collected specimens from the nose and nasopharynx for professional use in medical centers in EU countries.

## 2. Materials and Methods

### 2.1. Ethical Statements

The investigation was approved by the local Bioethics Committees (separately for each research center), the Medical University in Warsaw (Resolution No. KB/1382/22.), the Collegium Medicum of Nicolaus Copernicus University in Torun (Resolution No. KB/287/2022) and the Medical University in Bialystok (Resolution No. APK.002.140.2022) and was conducted in accordance with the World Health Medical Association 1964 Declaration of Helsinki and the EU rules of Good Clinical Practice.

### 2.2. Research Centers, Study Design, Patient Group and Sample Collection

The study was conducted at three research centers, two COVID laboratories listed by the Polish Ministry of Health—the National Medicines Institute (NMI) and the F. Lukaszczyk Oncology Center (OC)—and a supporting unit, the Medical University of Bialystok (MUB). At the first two sites, the trial was prospective and lasted from 4 April to 20 May 2022, while at the latter, it was retrospective using previously collected (February–March 2022) swabs of SARS-CoV-2 positive samples.

According to the study design, participants had to take three swabs from each patient, from the nasal cavity and nasopharynx for the tested antigen device and from the nasopharynx as a recommended specimen for the RT-qPCR reference method in both types of tested samples [22]. In order to meet the criteria of the approving authority, the study group had to consist of a minimum of 100 subjects with a positive RT-qPCR result for SARS-CoV-2 and 300 with a negative one [13].

All patients met the inclusion criteria, i.e., they were European adults and did not meet exclusion points, such as receiving a biologic drug or device covering treatment or therapy within the past 30 days, or eating, drinking or smoking 10 min prior to swabbing. They signed an informed consent to participate in the study and declared the onset of illness symptoms (fever, fatigue, dry cough, nasal congestion, runny nose, sore throat, myalgia and diarrhea) within the past week or no signs of infection.

The samples were collected by trained swabbers, and antigen tests were performed by scientists according to the kit manufacturer’s instructions. For the prospective investigation, RATs were carried out immediately after swab collection, whereas the reference method study was performed after collecting materials from all patients examined on that day. In cases where the test could not be performed immediately, on-site swabs were frozen (between −26 °C and −36 °C) immediately after sampling and then used in the retrospective study. The obtained specimens were properly coded so that all data would remain anonymous. The collection of positive SARS-CoV-2 samples represented naturally occurring viral loads without preselection.

### 2.3. Diagnostics Medical Device Candidate

GenBody COVID-19 Ag is an immunoassay assay for the rapid and qualitative determination of SARS-CoV-2 infection from nasal and nasopharyngeal swabs. The test cassette of the device contains monoclonal antibodies against the virus nucleocapsid protein. When the study sample contains SARS-CoV-2 antigens, the gold-conjugated anti-SARS-CoV-2 monoclonal antibodies bind to the SARS-CoV-2 antigens in the specimen and form antigen–antibody complexes. These complexes are captured by anti-SARS-CoV-2 monoclonal antibodies, which are immobilized and expressed as a line in the test zone. Unbound complexes further migrate out of the test region and are captured in the control area where they are imaged as a line (Figure 1). The appearance of a line in the control field is essential for the correct interpretation of the test. The technical parameters of the test were reported and verified during the analytical validation conducted by Kim et al. [23].

### 2.4. Reference Devices Description

A detailed overview of the devices and kits used in the study in the reference method is presented in Table 1. All RT-qPCR systems carried the European mark of conformity (CE), and the RT-qPCR reagents for the identification of SARS-CoV-2 infection were in vitro diagnostic medical devices. Both the test parameters and reading rules were performed according to the test manufacturer’s recommendations.

### 2.5. Performance Evaluation

RATs were evaluated by determining several values, such as positive/negative agreement and positive/negative predictive value, as well as a negative likelihood ratio, overall accuracy and positive co-incidence ratio.

The positive and the negative agreement of the assay (diagnostic sensitivity and specificity, respectively) were calculated as follows:

positive agreement (%) = A/(A + C) × 100 negative agreement (%) = D/(B + D) × 100

Positive and negative predictive values were calculated according to the formula:

positive predictive value (%) = [A/(A + B)] × 100 negative predictive value (%) = [(D/(D + C) ] × 100

In order to investigate the correspondence between the RATs result and clinical concepts of ruling out disease, the negative likelihood ratio was estimated as:

negative likelihood ratio (%) = [(1 − sensitivity)/specificity] × 100.

Overall accuracy was expressed as:

accuracy (%) = (A + D)/(A + B + C + D) × 100

For SARS-CoV-2 positive samples depending on the threshold cycle (CT) range, the co-incidence ratio was calculated as:

positive co-incidence ratio = E/F

The letters in the above formulas are assigned the following values:

- (True positive) constituted the number of positive samples in both developed and reference tests;
- (False positive) represented the number of positive samples in the developed test, but negative in the reference method,
- (False negative) were samples negative in the tested device and positive in the reference method;
- (True negative) were samples in both the tested device and reference method;
- Was the number of positive cases in the reference method based on the CT value;
- Referred to positive RAT results for a specific CT.

## 3. Results

### 3.1. Patient Group and Specimen Characteristics

The investigators were required to perform research following the described procedures. There were no protocol deviations. During the study, no sample was excluded, and no results were rejected. Sets of *n* = 303, *n* = 11 and *n* = 90 specimens were collected and tested at the NMI, the OC and the MUB, respectively. Finally, 404 samples were qualified for the investigation, including 103 positive and 301 negative cases confirmed by RT-qPCR tests. The study patient group consisted of *n* = 284 (70.3%) females, including *n* = 211 negative and *n* = 73 positive samples in RT-qPCR tests, and *n* = 120 (29.7%) males, including *n* = 90 and *n* = 30 negative and positive cases, respectively. They represented the age range from 18 to 95 years with a median age of 48 years. The most numerous group was participants aged 36–64 with *n* = 188 (46.53%) (*n* = 156 negative samples and *n* = 32 positives samples), followed by those aged 19–35 with *n* = 127 (31.44%) (*n* = 62 and *n* = 65 negative and positive results, respectively) and ≥ 65 with *n* = 89 (22.03%) (*n* = 83 negative and *n* = 6 positive cases).

All patients positive for SARS-CoV-2 by RT-qPCRs reported the same symptoms, such as fever, fatigue, runny nose, cough, headache, sore throat and body aches. In the group of negative patients, only six reported a runny nose. Detailed characteristics of the positive patients are shown in Table 2.

### 3.2. Medical Device Candidate Evaluation Results

The results obtained for the GenBody COVID-19 Ag nasal and nasopharyngeal RATs as IVD medical device candidates compared to the RT-qPCRs are summarized in Table 3. There were *n* = 6 (1.49%) false-negative results reported for the nasal specimen and only *n* = 3 (0.74%) for swabs taken from the nasopharynx.

Based on the data collected in Table 3, the test sensitivity was 94.17% for the nasal swab and 97.09% for the nasopharyngeal swab, and specificity was 100% for both samples. The accuracy of the test was 98.51% for nasal and 99.26% for nasopharyngeal swabs. The negative likelihood ratio was counted to determine whether a test result usefully decrease the probability that the patient had SARS-CoV-2 infection. The particular results of the validation parameters with a 95% confidence level are shown in Table 4.

Positive samples represented naturally occurring viral loads which were divided into four CT-dependent groups to better visualize the test possibilities. These results are summarized in Table 5. At high viral load (CT ≤ 25), the indications of the tested device are 100% consistent with the reference method, regardless of the type of material, while at low viral load (CT > 31), they are only 50% and 66.67% consistent for nasal and nasopharyngeal swabs, respectively. The positive co-incidence rate, as the probability of the occurrence of an identical result in both the test and reference method, was 1 only for specimens with a high number of RNA copies (CT **≤** 25, and CT between 25 and 28 only for nasopharyngeal swab).

## 4. Discussion

Products in regulated fields such as medical devices must comply with strict quality standards throughout the entire process of production, be safe and meet their intended use. Verification, analytical and clinical validation processes should be applied to all devices used in medicine and must also comply with applicable good practice guidelines, e.g., manufacturing or laboratory. It is important to develop clear study protocols and report templates before starting the process. Verification documentation should stipulate the acceptance criteria, testing steps, procedures and results documentation with relevant conclusions, while analytical and clinical validation processes are governed by the regulations applicable to human experimentation. Clinical trial protocols are required by regulatory agencies and are scientific evidence that determines whether a given technology is appropriate for the intended use and application context. It is sometimes difficult to draw a clear line between verification, analytical and clinical validation, and the terms are often used interchangeably or to describe the evaluation of test performance (sensitivity and specificity) in a clinical application [24,25,26,27]. Evaluation in clinical application and clinical validation in clinical trials differ not only in the purpose of using the results but also in the study design. Different entities are involved in the implementation of the study, e.g., engineers, clinical researchers from various fields of medicine and regulatory agencies specialists, so it is extremely important to clarify the core terminology and best practices for the evaluation of medical devices [11].

Each IVD medical device must be marketed in accordance with the guidelines, which include, among other things, a certificate confirming the safety of the test and all information regarding its performance evaluation [13,14]. Analytical validation of the GenBody COVID-19 Ag, including the limit of detection, as well as an assessment of specificity based on the lack of reaction to any related beta-coronaviruses, and clinical validation on an Asian population was previously performed by Kim et al. [23]. Here, we conducted the first clinical validation of these assays on a European population with the aim of introducing them to the EU COVID-19 common diagnostic test list. Prior research conducted in Europe only examined archival material in a retrospective survey. Our study focused mostly on prospectively collected specimens and thus expanded the current recommendations for the tests. Sample size and their selection complied with the document for IVD medical devices and the EU regulations for a common list of COVID-19 rapid antigen tests [13,14]. However, some authors point out that in the selection of the target group, it is important to take into account parameters such as assumptions for power, error and prevalence [28,29]. The size of the study group in our investigation was sufficient to conclude the test parameters.

No adverse reactions occurred in patients during the trial, and no samples were excluded. There were no false positives in either nasal or nasopharyngeal swabs, so the specificity and positive predictive value of the candidates for medical devices was 100%. A high positive predictive value means that a patient with a positive test result is highly likely to be infected with the virus or, on the other hand, a positive test does not require verification by other methods [30]. This value should be interpreted with caution as it depends on the prevalence of the disease in the population [28,29,31], whereas the specificity shows the test’s ability to correctly reject healthy patients without a condition [30]. According to the European Directorate General for Health and Food Safety guidelines, only a specificity above 98% for the rapid test and 99% for the laboratory assay meets acceptance directives [13,14]. The high specificity of GenBody COVID-19 Ag tests was also obtained by researchers who conducted studies (nasopharyngeal or pharyngeal swabs) in an Asian population [23,32]. In our study, we confirmed the data from Korea and additionally showed that nasal samples also show 100% specificity, and these swabs for testing are easier to collect. The opposite results were obtained by researchers from Hungary, where, in a group of 98 patients (nasopharyngeal swabs), the specificity of GenBody COVID-19 Ag was 90% (95%CI 79–96%) [33]. However, this examination was not carried out for the purpose of registering a medical device, so the study design did not have to meet the criteria of the approval authority. Importantly, the value of the negative likelihood ratio allows stating that a negative test result significantly reduces the probability of the disease, which is key information for a clinician in the diagnostic process.

In our investigation, the average sensitivity of the GenBody COVID-19 Ag test was calculated above 95% and was higher for the nasopharyngeal swab compared to the nasal swab, meeting the criteria of the EU guidelines, which are above 80% for the rapid test and 85% for the laboratory test [13,14]. Authors from Korea and the Netherlands also noted the high sensitivity of this RAT [23,34]. In contrast to these results, in the studies of the India and Hungary teams, the test showed very low overall sensitivity but was already high for samples with low CT values [32,33]. Thus, the probability of a positive result in both the GenBody COVID-19 Ag test and the reference method is higher when CT is lower, as indicated by the co-incidence index. It is worth adding that the clinical sensitivity of the test increases with the number of virus copies in the sample expressed as CT, which may be related to the limit of detection [23,33,35]. The CT values may vary depending on the used protocol/reagents, as well as which part of the SARS-CoV-2 genome is targeted. The assays used in this trial showed a similar correlation between the detected number of viral gene copies (gc) and the CT value and amounted to over 10^5^ gc/mL, 10^5^–10^4^ gc/mL, 10^4^–10^3^ gc/mL and under 10^3^ for CT ≤ 25, 25 < CT ≤ 28, 28 < CT ≤ 31 and CT > 31, respectively [36]. The coronavirus variant can also have a significant impact on the sensitivity of the medical device, which was observed by Hagag et al. [37]. The authors showed that mutations in the SARS-CoV-2 nucleocapsid can lead to a reduction in test sensitivity through failure to detect viral antigens or partially recognizing them, leading to reduced test line intensity. Furthermore, some researchers believe that thawing the sample reduces the stability of the RNA which may increase the CT value in RT-qPCR and give false-negative results in the antigen test [38]. Other studies, including our own (unpublished data), have shown that the effect of freezing and thawing samples on CT values and test line intensity in RAT is clinically insignificant [39,40]. In our investigation, we observed only a few false-negative results; three for nasopharyngeal samples (a single for CT between 29 and 31 and two for CT > 31) and six for nasal specimens (a single for CT between 26 and 28, two for CT between 29 and 31 and three for CT > 31).

A limitation of the study was that the genomes of SARS-CoV-2 causing the infections were not sequenced, so we do not know which variants of the virus were identified. However, it is reasonable to assume from national data (February-May 2022) that it was most likely the Omicron variant, which appeared in Poland in December 2021 and until now is the most commonly detected virus type [41,42,43].

## 5. Conclusions

The presented study was a clinical validation of the GenBody COVID-19 Ag RATs conducted for the first time on Europeans. The test using nasopharyngeal swabs had previously only been clinically validated on the Asian population. In our study, in addition to nasopharyngeal samples, nasal samples were examined for the first time. In both cases, the tests were safe to use and showed high sensitivity and specificity.

## Figures and Tables

**Table 1:** Reagents and systems used in the reference method depending on site.

	NMI	OC	MUB
Nucleic acid extraction	NucleoMag Pathogen kit	3DMed Sample Transport Medium (Extraction free)	MagSi-Na Pathogens kit PurePrep 96 Nucleic Acid Purification System
	Macherey-Nagel Duren, Germany	3D Biomedicine Science & Technology Shanghai, China	Magtivio Nuth, Netherlands
RT-PCR system	QuantStudio™ 6	QuantStudio™ 5	CFX96™ Dx
	Life Technologies Singapore	Life Technologies Singapore	Bio-Rad California, CA, USA
RT-PCR kit	MutaPLEX^®^ Coronavirus	ANDiS FAST SARS-CoV-2 RT-qPCR Detection Kit	Allplex SARS-CoV-2 fast PCR Assay CE IVD
	Immundiagnostik AG Bensheim, Germany	3D Biomedicine Science & Technology Shanghai, China	Seegene Seoul, Republic of Korea

**Table 2:** Characteristics of SARS-CoV-2 positive patient group by age, gender and CT values obtained in RT-qPCRs.

Age	Gender *	CT Range			
		CT ≤ 25	25 < CT ≤ 28	28 < CT ≤ 31	CT > 31
19–35	F	29	12	5	2
	M	9	5	2	1
36–64	F	9	7	0	3
	M	5	4	4	0
>65	F	5	0	1	0

* F—female, M—male.

**Table 3:** GenBody COVID-19 Ag test results compared to reference method in nasal and nasopharyngeal samples.

Specimen	Candidate Device	RT-PCR	
		Positive	Negative
Nasal swab	Positive	97	0
	Negative	6	301
Nasopharyngeal swab	Positive	100	0
	Negative	3	301

**Table 4:** Validation parameters for GenBody COVID-19 Ag RATs.

GenBody COVID-19 Ag Test Result	Specimen			
	Nasal Swab		Nasopharyngeal Swab	
Sensitivity	94.17%	(95%CI 87.75% to 97.83%)	97.09%	(95%CI 91.72% to 99.4%)
Specificity	100%	(95%CI 98.78% to 100%)	100%	(95%CI 98.78% to 100%)
Positive predictive value	100%		100%	
Negative predictive value	98.05%	(95%CI 95.85% to 99.09%)	99.01%	(95%CI 97.05% to 99.67%)
Negative likelihood ratio	0.06	(95%CI 0.03 to 0.13)	0.03	(95%CI 0.01 to 0.09)
Accuracy	98.51%	(95%CI 96.80% to 99,45%)	99.26%	(95%CI 97.85% to 99.85%)

**Table 5:** Positive samples (determined by the reference method) divided according to the CT compared to GenBody COVID-19 Ag RATs in nasal and nasopharyngeal swabs.

CT Range	CT ≤ 25	25 < CT ≤ 28	28 < CT ≤ 31	CT > 31
No. of positive cases in RT-qPCR	57	28	12	6
Nasal swab No. of positive cases in tested device	57	27	10	3
Nasopharyngeal swab No. of positive cases in tested device	57	28	11	4
Nasal swab Sensitivity of the test depending on CT	100% (95%CI 93.73% to 100%)	96.43% (95%CI 81.65% to 99.91%)	83.33% (95%CI 51.59% to 97.91%)	50% (95%CI 11.81% to 88.19%)
Nasopharyngeal swab Sensitivity of the test depending on CT	100% (95%CI 93.73 to 100%)	100% (95%CI 87.66% to 100%)	91.67% (95%CI 61.52 to 99.79%)	66.67% (95%CI 22.28% to 95.67%)
Positive co-incidence rate *	1/1	0.96/1	0.83/0.92	0.5/0.67

* The values are separated for nasal and nasopharyngeal swabs.

## Data Availability

All relevant data are within the manuscript.
